# Prostate cancer metastasis and soy isoflavones: a dogfight over a bone

**Published:** 2019-02-19

**Authors:** Vladimir Ajdžanovic, Branko Filipovic, Dragana Miljic, Sanja Mijatovic, Danijela Maksimovic-Ivanic, Marko Miler, Jasmina Živanovic, Verica Miloševic

**Affiliations:** 1 Department of Cytology, Institute for Biological Research "Siniša Stankovic", University of Belgrade, Belgrade, Serbia; 2 Clinic for Endocrinology, Diabetes and Diseases of Metabolism, Clinical Center of Serbia, Faculty of Medicine, University of Belgrade, Belgrade, Serbia; 3 Department of Immunology, Institute for Biological Research "Siniša Stankovic", University of Belgrade, Belgrade, Serbia

**Keywords:** prostate cancer, metastasis, bones, soy isoflavones

## Abstract

Prostate cancer is a complex, progressive, bone-tropic disease, which is usually associated with skeletal issues, poor mobility and a fatal outcome when it reaches the metastatic phase. Soy isoflavones, steroid-like compounds from soy-based food/dietary supplements, have been found to decrease the risk of prostate cancer in frequent consumers. Herein, we present a systematization of the data on soy isoflavone effects at different stages of metastatic prostate cancer progression, with a particular interest in the context of bone-related molecular events. Specifically, soy isoflavones have been determined to downregulate the prostate cancer cell androgen receptors, reverse the epithelial to mesenchymal transition of these cells, decrease the expressions of prostate-specific antigen, matrix metalloproteinase and serine proteinase, and reduce the superficial membrane fluidity in prostate cancer cells. In addition, soy isoflavones suppress the angiogenesis that follows prostate cancer growth, obstruct prostate cancer cells adhesion to the vascular endothelium and their extravasation in the area of future bone lesions, improve the general bone morphofunctional status, have a beneficial effect on prostate cancer metastasis-caused osteolytic/osteoblastic lesions and possibly affect the pre-metastatic niche formation. The observed, multilevel antimetastatic properties of soy isoflavones imply that they should be considered as promising components of combined therapeutic approaches to advanced prostate cancer.

## Introduction

Prostate cancer is the most common cancer in men worldwide, manifesting considerable racial, ethnic, geographic and socioeconomic status-related differences in its incidence and mortality (Rebbeck, 2017[108]; Pernar et al., 2018[105]; Kimura and Egawa, 2018[71]). The highest age-adjusted incidence rates were observed in developed countries (the African-American male population in the United States is the most vulnerable in this respect), while the lowest prostate cancer incidence is characteristic of Asian men living in their native countries (Rebbeck, 2017[108]; Pernar et al., 2018[105]). On the other hand, Afro-Caribbean and Sub-Saharan African populations have the highest prostate cancer mortality rates, strongly correlated with limited access to medical care, *i.e.* prostate-specific antigen (PSA)/ultrasound screening and the possibility of early detection of the disease, or to adequate therapy (Rebbeck, 2017[108]; Pernar et al., 2018[105]). Predominance of an androgen-independent cell phenotype in the prostate tumor is one of the crucial moments in the malignant disease progression and brings bad news for the patients (Tang and Porter, 1997[127]; Arnold and Isaacs, 2002[14]). In parallel, 'bone tropism' or the preference of prostate cancer cells for bone invasion and colonization, resulting from a sequential series of targetable molecular events, underlies the decreased quality of life, skeletal pain/complications and mortality of these cancer patients (Rucci and Angelucci, 2014[113]; Ziaee et al., 2015[148]). The metastasizing of prostate cancer cells to bones is a microenvironment-adjusted process, considering the cancer cell-bone tissue cross-talk, and involves numerous signaling pathways (Jin et al., 2011[63]; Ziaee et al., 2015[148]). The poor prognosis of a prostate cancer in its metastatic stage suggests the need for improving the available diagnostic methods as well as for finding innovative approaches to establishing a safe and promising therapeutic strategy. The existing treatment protocols and guidelines regarding prostate cancer highlight a number of factors that should be considered during therapy, such as: existence of concrete symptoms, serum androgen and PSA levels, type of metastasis if present (bone/visceral), treatment history, performance status, side effects of the therapy, etc. (Crawford et al., 2015[30]). In line with this, androgen deprivation therapy (ADT) is the treatment of choice and has a high response rate in the early stages of the disease, while the options available for treating metastatic castration-resistant prostate cancer (with still activated but deviant androgen receptor (AR) signaling) may include AR-targeted therapy (abiraterone, enzalutamide), chemotherapy (docetaxel and cabazitaxel), immunotherapy (sipuleucel-T), bisphosphonates or radionuclides (radium-223) (Grossmann et al., 2001[46]; Nuhn et al., 2019[98]). Palliative care for patients suffering from metastatic prostate cancer is a challenging task that requires a multimodal therapeutic approach (Das and Banerjee, 2017[32]).

In recent decades, interest in the plant-derived compounds relevant for cancer prevention and therapy has increased substantially. Soy isoflavones are steroid-like (the chemical features of these compounds have been more thoroughly described in our previous works - Ajdžanović et al., 2012[5]; 2014[9]; 2018[8]), non-nutrient components of soy-based food and therapeutic dietary supplements whose application is *inter alia* associated with improved bone health in both normal and osteoporotic male rodents (Chin and Ima-Nirwana, 2013[26]) as well as with low risk of prostate cancer, especially in frequent consumers such as Asian-Pacific men (Messina, 2010[89]; Ajdžanović et al., 2014[9]; Mahmoud et al., 2014[86]; Sak, 2017[115]; Xiao et al., 2018[136]). There is now a growing body of evidence on the exact mechanisms by which these compounds of natural origin may prevent the development or progression of prostate cancer (Mahmoud et al., 2014[86]). It would be too ambitious to compare the specificity and therapeutic potential of soy isoflavones with those of the newly developed pharmacotherapeutics; however, since isoflavones have been well recognized as bone-modifying agents (Messina, 2010[89]; Messina et al., 2010[90]; Filipović et al., 2010[40], 2018[41]; Chin and Ima-Nirwana, 2013[26]; Zheng et al., 2016[146]), we believe that, in the specific context of prostate cancer bone metastasis formation, the effects of their application deserve some attention. Here, we will focus on the potential role of soy isoflavones in the prevention and treatment of bone metastasis in prostate cancer, as very important aspects of this cancer management.

## A Brief Overview of the Mechanisms of Bone Metastasis in Prostate Cancer

Prostate cancer, like most other solid tumors, shows an intrinsic tendency toward metastasizing to distant organs (lungs, liver, brain), but it has a pronouncedly high preference for metastasizing to the bone (vertebrae, femur, pelvis, ribs; Yang et al., 1999[139]; Jin et al., 2011[63]). This principle of malignant disease spreading, with secondary deposits formation, is metaphorically presented in the 'seed and soil' model (Paget, 1889[101]), where cancer cells or 'seeds' metastasize to the 'soil' most appropriate for their growth. Actually, the bone contains chemotactic factors that attract prostate cancer cells and direct their movement. Stromal-derived factor-1 (SDF-1), epidermal growth factor (EGF), insulin-like growth factor (IGF), hepatocyte growth factor (HGF), Type I collagen, osteonectin and bone sialoprotein have been shown to act as chemoattractants for prostate cancer cells, predominantly causing them to gravitate towards the bone (Jacob et al., 1999[60]; Taichman et al., 2002[125]; Stewart et al., 2004[123]; Arya et al., 2006[15]). More broadly, the metastasizing of prostate cancer is a multistep process that implies angiogenesis at the primary site, loss of the cancer cells' adhesion, followed by their local migration, intravasation into the vasculature or lymphatics, transport *via* circulation, extravasation, and homing to distant organs, which is again followed by the angiogenesis step (Arya et al., 2006[15]). Interestingly, recent findings have suggested the existence of an early pre-metastatic phase, preceding the homing of cancer cells to the bone tissue (Gartland et al., 2016[43]). This formation of a pre-metastatic niche could be described as an adaptation of an apartment for the arrival of a new occupant. The discovery of pre-metastatic niches confirms the impressive communication between primary sites and distant tissues, selected for dissemination according to criteria that are still unclear. It has become obvious that this step is navigated by products present in the primary tumor microenvironment, the so-called cancer secretome, which is responsible for the formation of a pre-metastatic zone in the particular distant organ (Gartland et al., 2016[43]).

A brief summary of the most important mechanisms relevant for prostate cancer metastasis to bone is given below.

### The role of androgen receptors (ARs)

Androgens, realizing their actions through ARs, have a crucial role in prostate cancer development and progression, most likely at all stages of the disease (Cunha et al., 2004[31]; Jin et al., 2011[63]; Ziaee et al., 2015[148]). The cascade of molecular events implies androgen binding to the AR and translocating to the nucleus, where the binding of this complex to androgen responsive elements occurs, which affects the expression of various genes. As a result, proliferation of prostate cancer cells is favored at the expense of their apoptosis (Jin et al., 2011[63]). ADT (chemical or surgical castration) is the treatment of choice for this malignant disease in its early stages. As previously indicated, the prostate cancer acquiring a castration-resistant phenotype is the inevitable next stage of the disease, which relies on the following events that take place in the cancer cells: upregulation and mutation of ARs with irregular downstream gene expression, altered expression and function of AR coactivators, ligand independent AR activation, increased autocrine and paracrine production of androgens and elevated expression of interleukin-6 (Adler et al., 1999[2]; Chen et al., 2000[25]; Debes and Tindall, 2004[35]; Linja et al., 2004[82]; Dutt and Gao, 2009[37]; Bonkoff and Berges, 2010[20]; Jin et al., 2011[63]; Mahmoud et al., 2014[86]).

### Insight into the epithelial to mesenchymal transition (EMT) and adhesiveness of prostate cancer cells

The adhesiveness of prostate cells decreases if they 'move along the way' of malignant transformation (Jin et al., 2011[63]; Jadaan et al., 2015[61]). Namely, in the process of epithelial to mesenchymal transition (EMT), essential for the development of a more invasive cancer cell phenotype, the static, polarized epithelial cells transform into migratory, spindle-shaped mesenchymal cells (Thiery, 2002[130]; Yang and Weinberg, 2008[138]; Jadaan et al., 2015[61]). The EMT, which is specific for higher grades of prostate cancer, is accompanied by cadherin protein switching, which includes the downregulation of E-cadherin (characteristic of normal epithelial cells) and upregulation of N-cadherin (abundant in mesenchymal cells) (Gravdal et al., 2007[45]). At this stage of prostate cancer progression, the expression of β-catenin also decreases (Jaggi et al., 2005[62]), all of which contributes to the loosening of connections between the cells, given the important role of E-cadherin and β-catenin complexes in the maintenance of cell-cell adhesions (Jin et al., 2011[63]). In parallel, intrinsic overexpression of miRNA-409 and downregulation of miRNA-143 and -145 promote the EMT of prostate cancer cells and support the shaping of their metastatic phenotype (Peng et al., 2011[104]; Josson et al., 2014[66]). Of note, somewhat higher expressions of E-cadherin and β-catenin have been reported in the metastatic prostate cancer cells that have already reached the bone (Saha et al., 2008[114]), suggesting that reverse, mesenchymal to epithelial transition (MET) is a prerequisite for the growth of metastatic cells at the site of a secondary bone deposit (Jin et al., 2011[63]). Focal adhesions, or more precisely the macromolecular complexes mediating the extracellular matrix (ECM)-cell cytoskeleton contacts, convert physical vectors into chemical signaling, thus affecting the cell's dynamic properties (Bershadsky et al., 2003[19]; Ajdžanović et al., 2014[9]). Variable expression of transmembrane integrin proteins, regulators of focal adhesions, and the downstream signaling molecules they affect, is associated with decreased adhesion of prostate cancer cells and metastasis (Hao et al., 1996[51]; Slack-Davis and Parsons, 2004[121]; Nicolas and Safran, 2006[97]). Overexpression of focal adhesion kinase (FAK) and the Src family of kinases, the nonreceptor tyrosine kinases that are the crucial signaling molecules outputting focal adhesions, is characteristic of a migratory metastatic prostate cancer cell phenotype (Rovin et al., 2002[111]; Kim et al., 2009[70]; Tatarov et al., 2009[129]). Given the fact that increased membrane fluidity enhances the malignancy of cancer cells *in vitro* (Zeisig et al., 2007[142]) and correlates with the decreased cancer cell adhesiveness (Gonda et al., 2010[44]), we will prove that the superficial membrane fluidity of LNCaP and PC-3 prostate cancer cells (isolated from lymph node and bone metastasis, respectively) at least partially determines their invasive activity (Ajdžanović et al., 2013[6], 2014[9]), therefore completing the previous observations in this context.

### Degradation of the extracellular matrix (ECM) integrity by prostate cancer cells

Prostate cancer invasion and metastasis require partial degradation of the ECM integrity. This process is mediated by families of proteinase enzymes, such as matrix metalloproteinases (MMPs), serine proteinases (urokinase-type plasminogen activator-uPA and plasmin), and probably by PSA, generally known as a fibronectin-degrading proteinase (Jin et al., 2011[63]). In human prostate cancer tissues, upregulation of MMPs correlates with the loss of tissue inhibitor of MMP-1 (Brehmer et al., 2003[22]), the metastatic phenotype brings high plasma concentrations of MMP-2 and MMP-9 (Morgia et al., 2005[93]), while MMP-12 participates in bone-tropic metastasis (Nabha et al., 2008[94]). A biomechanical point of view suggests that MMPs activity facilitates the expansion tendency of prostate cancer cells and their navigation through the 'cracks' of the degraded matrix (Ajdžanović et al., 2013[6], 2014[9]). A similar pattern of ECM degradation characterizes the invasive prostate cancer cell-specific activity of uPA, the proteinase simultaneously involved in the activation of latent MMPs and conversion of plasminogen into the functional, matrix-degrading enzyme plasmin (Sheng, 2001[118]; Arya et al., 2006[15]).

### Circulation- and bone-related aspects of prostate cancer cell dissemination

Intravasation of metastatic prostate cancer cells and their entry into the circulation impose the need for survival in a new milieu, and precede the adhesion of malignant cells to the vascular endothelium and extravasation into bone. Vascular endothelial growth factor (VEGF) and its receptor (VEGFR), as potent stimulators of angiogenesis, are highly expressed in prostate cancer (Pallares et al., 2006[102]). Recruited and organized endothelial cells provide a blood supply that nourishes the prostate cancer mass and facilitate the cancer cell metastasis at distant loci (Ziaee et al., 2015[148]). The membrane fluidity of freely circulating cancer cells is found to be more than double that of the cells adhering to the inner vascular surface, and around 23 times higher than the membrane fluidity of already adhered cancer cells, migrating over the vascular surface (Gonda et al., 2010[44]). A 'dock and lock' mechanism has been proposed for the instance of cancer cells binding to the vascular endothelium (Honn and Tang, 1992[55]). The adhesion molecule P-selectin, expressed by the bone endothelial cells, and sialyl-Lewis^x^ carbohydrate, available on the prostate cancer cell surface, play a crucial role in the association (Martensson et al., 1995[87]; Mazo and von Andrian, 1999[88]), while integrin molecules mediate the subsequent locking process (Romanov and Goligorsky, 1999[110]). In addition, prostate cancer cell- and osteoclast-integrin and cancer cell/osteoblast cadherin 11 molecules appear to be important in the colonization of these malignant cells in the bone (Chu et al., 2008[28]; Jin et al., 2011[63]). Growth of prostate cancer cells settled in the bone is accompanied by their production of growth factors that stimulate proliferation and maturation of osteoblasts and osteoclasts, and the release of these factors in turn stimulates metastatic growth (the 'vicious cycle'; Jin et al., 2011[63]). The inevitable disbalance between bone formation and bone resorption, characteristic of metastatic prostate cancer, results in osteoblastic or osteolytic lesions (Ibrahim et al., 2010[57]). Osteoblastic lesions with irregular, increased bone formation occur more frequently (Urwin et al., 1985[134]) and represent hot spots for bone fractures. Formation of osteolytic lesions, on the other hand, releases a three-dimensional space for further prostate cancer metastasis progression and at the same time liberates the molecules involved in further bone and tumor cell proliferation. These findings complete the pool of data pertinent to dysregulated osteoblast and osteoclast function in metastatic-related bone remodeling. The bone aspect, both initially and at the later stages of prostatic cancer disease, will determine the diagnostic procedures, treatment, complications, quality and duration of life of the patients (Butoescu and Tombal, 2014[23]).

### The phenomenon of pre-metastatic niche formation: general and prostate cancer-specific considerations

Does the metastatic process start before detectable cancer cells appear in the bone tissue?

As mentioned above, the reposition of circulating tumor cells (CTCs) into distant organs is crucial for the development of metastatic foci. Obviously, this step is critically affected by the local microenvironment at the place of CTC arrival. Sound data have recently suggested that a primary tumor can prearrange its new home before a cancer cell enters the site by inducing a supportive microenvironment recognized as a pre-metastatic niche (Kaplan et al., 2005[68]; Liu and Cao, 2016[84]). The pre-metastatic niche could be described as an area where fine accommodation for the newcomer has been prepared, offering all the conditions for colony formation, in close interaction with the surrounding tissue.

Kaplan et al. (2005[68]) showed that bone marrow-derived hematopoietic progenitor cells (BMDC) settle at tumor-specific pre-metastatic sites and form cellular clusters before the arrival of tumor cells. Those cells express vascular endothelial growth factor receptor 1 (VEGFR1), and depletion of VEGFR1^+^ cells from the bone marrow of wild-type mice disables the formation of pre-metastatic clusters and prevents metastasis development, thus confirming the hypothesis of their role in the process of dissemination. Since a functional reconstitution of this cell population in Id3 (inhibitor of differentiation 3) knockout mice resulted in the reestablishment of the entire process, from cluster formation to the development of metastasis, it is clear that those cells represent the key actors in pre-metastatic niche formation (Kaplan et al., 2005[68]). Until today, a long list of molecules involved in pre-metastatic niche formation has been defined. Among the niche promoting molecules are TGF-β (Hiratsuka et al., 2008[53]; Olkhanud et al., 2011[100]), RANK/RANKL (Chu et al., 2014[27]), Lysil Oxidase enzyme (LOX) (Erler et al., 2009[38]), Hypoxia Inducible Factors (HIFs) (Unwith et al., 2015[132]), etc. LOX enzyme-mediated remodeling of ECM in the bones leads to the formation of a pre-metastatic niche within the bone microenvironment, favoring the homing of CTC and subsequent development of dissemination lesions (Cox et al., 2015[29]). Data about the pivotal role of LOX proteins in the development of prostate cancer metastasis has been recently provided. Crosslinking of collagen, conducted with the LOX derived from stromal cells, was shown to control the movement of prostate cancer cells, while LOX inhibition obstructed the same process (Caley et al., 2016[24]). Interestingly, LOX pro-peptide (LOX-PP), as an intermediary product in the formation of the final form of LOX enzyme, functions as a tumor suppressor (Trackman, 2016[131]). Despite its opposite role in comparison with the mature protein counterpart, in the case of intramedullary injections of PC-3 LOX-PP expressing prostate cancer cell lines, enhanced appearance of osteolytic lesions and subsequent bone destruction were discovered *in vivo* (Alsulaiman et al., 2016[13]). It is important to underline that the concept of a pre-metastatic niche perfectly fits into both the linear and parallel models of metastasis development.

### Osteolytic prostate cancer metastases

Presence of specific bone metastases that coincide with osteolytic lesions implies a pronounced participation of the receptor activator of nuclear factor-κB (RANK)/RANK ligand (RANKL)/osteoprotegerin (OPG) axis (Jin et al., 2011[63]). RANKL, regularly produced by osteoblasts, can also be the secretory product of metastatic prostate cancer cells that directly activates osteoclasts *via* RANK (Zhang et al., 2003[143]). The mentioned process of osteoclast activation and bone resorption is inhibited by OPG derived from osteoblasts; however, this molecule simultaneously protects cancer cells from apoptosis (Holen et al., 2002[54]; Boyle et al., 2003[21]), thus expressing its antinomic nature. In this respect, parathyroid hormone-related protein (PTHrP), a homolog of parathyroid hormone, upregulates RANKL production in osteoblasts and decreases the OPG expression, all of which leads to the activation of osteoclasts and osteolytic metastasis formation (Liao et al., 2008[81]). Given that PTHrP also induces differentiation of osteoblasts (Liao et al., 2008[81]), its role in osteoblastic lesion formation appears certain.

Osteoclast-derived proteinases and prostate cancer cells-secreted PSA and uPA activate transforming growth factor β (TGF-β), which may also promote osteolytic metastases (Josson et al., 2010[66]; Jin et al., 2011[63]) through induction of the proosteolytic gene expression in cancer cells, with PTHrP in the key position (Yin et al., 1999[141]; Kingsley et al., 2007[72]). PC-3 cells, established from androgen independent prostate cancer bone metastasis, are potent producers of PTHrP prometastatic protein (Kingsley et al., 2007[72]). In addition, TGF-β is one of the important factors involved in EMT, whereby the PI3K/Akt pathway plays a noticeable role in this respect (Nakazawa and Kyprianou, 2017[95]). All together, the bone matrix (containing calcium, TGF-β, insulin-like growth factors (IGF) I and II, etc.) with its physical features (low oxygen, local acidity) favors tumor progression and the subsequent formation of new osteolytic lesions, forming an amplification loop (Kinsley et al., 2007[72]).

### Osteoblastic prostate cancer metastases

Osteoblastic lesions are the main type of bone abnormalities in prostate cancer metastasis (Kingsley et al., 2007[72]). Dysregulated osteoblast activities are mediated by numerous 'osteoblastic' factors. Endothelin-1 (ET-1) is a vascular endothelium-produced vasoconstricting peptide of small size that increases osteoblast proliferation and initiates bone matrix formation (Yin et al., 2003[140]; Guise et al., 2003[47]). Thus, a diagnosis of osteoblastic prostate cancer metastasis implies elevated plasma levels of ET-1 (Nelson et al., 1995[96]). Proliferation of osteoblasts is also stimulated by Wnt signaling through β-catenin-induced gene expression (Behrens et al., 1996[16]). It should be mentioned that metastatic prostate cancer cells are also capable of producing Wnt proteins and stimulating osteoblast proliferation (Hall et al., 2006[49]), and therefore irregular bone formation. Osteoblasts produce bone morphogenic protein 2 (BMP-2) that can activate Akt, ERK and NFκB signaling, essential for the migration of prostate cancer cells (Lai et al., 2008[74]). Finally, IGF I is upregulated in prostate cancer bone metastasis and stimulates the cancer cell proliferation, while elevated levels of IGFs may coincide with osteoblast proliferation and the following susceptibility to bone fractures (Rubin et al., 2004[112]; Jin et al., 2011[63]).

## Prostate Cancer Metastases and Soy Isoflavones Application – the Bone Endpoints

In the next section of this analytical article, we will focus on prostate cancer metastasis formation in connection with the application of soy isoflavones. This up-to-date report attempts to elucidate the effects of these steroid-like compounds along the sequence of events relevant to the metastatic process, which culminates in the bones. A detailed mechanistic overview of the soy isoflavone actions, which may be useful for comprehending the concrete topic, can be found in our previous review articles (Ajdžanović et al., 2014[9], 2015[4]).

### Soy isoflavone effects on the AR signaling

**Figure 1:**
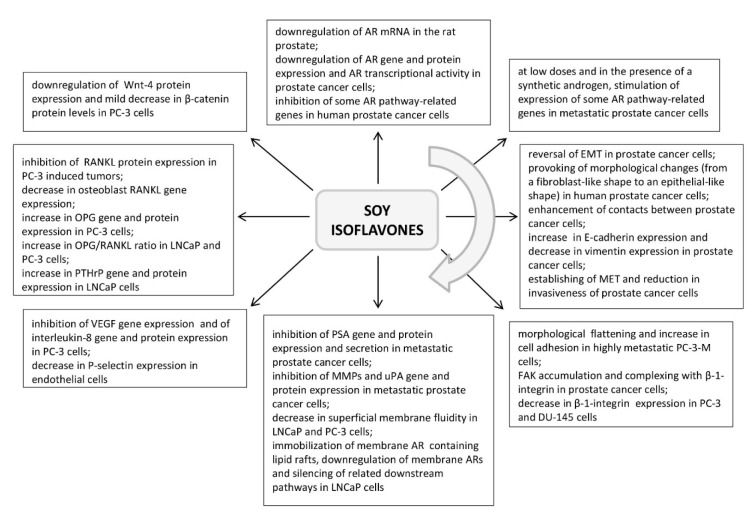
Highlights of the effects of soy isoflavones application along the sequence of events relevant to metastasis formation in prostate cancer (sorted in a clockwise direction; references are provided in the appropriate section of the article). ARs - androgen receptors, EMT - epithelial to mesenchymal transition, FAK - focal adhesion kinase, MET - mesenchymal to epithelial transition, MMPs - matrix metalloproteinases, OPG - osteoprotegerin, PSA - prostate-specific antigen, PTHrP - parathyroid hormone-related protein, RANKL - receptor activator of nuclear factor-κB ligand, uPA - urokinase-type plasminogen activator, VEGF - vascular endothelial growth factor

It has been reported that the soy isoflavone genistein, as a component of diet in concentrations comparable to the human intake (250 or 1000 mg/kg diet, 2 weeks), downregulates AR mRNA in the rat prostate (Fritz et al., 2002[42]). In prostate cancer cells, genistein (at 30 and 50 μM concentrations, 24 h) was shown to downregulate the AR gene and protein expression* in vitro*, as well as the receptor transcriptional activity (Davis et al., 2002[33]). A daidzein metabolite equol (50 μM, 48 h) was also found to suppress AR expression in LNCaP prostate cancer cells (Itsumi et al., 2016[59]). Furthermore, significant inhibition of some AR pathway-related genes in human prostate cancer cells was identified after genistein application (at 1 μM, 5 μM and 25 μM; 48 h) (Takahashi et al., 2004[126]) (Figure 1(Fig. 1)). The proposed mechanism of genistein action in this respect implies its binding to estrogen receptor β (ER-β) and the initiation of a cascade of molecular events resulting in the AR downregulation (Bektic et al., 2004[17]). However, some *in vitro* studies have demonstrated that genistein, at low doses (2 μM, 24 h) and in the presence of a synthetic androgen, may have a stimulating effect on the expression of AR pathway-related genes (PSA, KLK4, NKX3.1, STAMP2) in metastatic prostate cancer cells (Lazarevic et al., 2008[76]) (Figure 1(Fig. 1)). This phenomenon could at least partly be explained by the AR mutations associated with prostate cell malignant transformation and the ARs acquiring the capability to interact with a wide range of steroid-like compounds (Mahmoud et al., 2014[86]).

### EMT and adhesiveness of prostate cancer cells upon soy isoflavones application

The EMT of prostate cancer cells can be reversed with the active participation of soy isoflavones (Mahmoud et al., 2014[86]). Namely, culturing of IA8-ARCaP cells with low dosed genistein (15 μM/L for 24 h) morphologically changed these human prostate cancer cells, from a fibroblast-like shape to an epithelial-like shape (Zhang et al., 2008[144]). Incubation with genistein that lasted 48 h led to enhanced cell-cell contacts in this context (Zhang et al., 2008[144]) (Figure 1(Fig. 1)). The authors observed that genistein markedly increased the expression of E-cadherin and significantly decreased the expression of the mesenchymal marker vimentin, when applied in low doses to IA8-ARCaP as well as LNCaP/HIF-1a prostate cancer cells (Zhang et al., 2008[144]). By establishing a balance between the expressions of epithelial and mesenchymal protein markers which is actually characteristic of MET, genistein decreases the invasiveness of prostate cancer cells (Figure 1(Fig. 1)). Although the soy isoflavones, genistein and daidzein (at concentrations of 40 μM and 110 μM, 48 h), may affect the expression of certain miRNAs in prostate cancer cell clones (Rabiau et al., 2011[106]), to the best of our knowledge, their concrete effects on miRNA-409, -143 and -145 (responsible for the EMT and affirmation of a metastatic phenotype in prostate cancer cells) still remain unknown. Morphological flattening of highly metastatic prostate cancer PC-3-M cells upon genistein (50 μM, 2 h - 3 days) treatment was found to be accompanied by an increase in cell adhesion (Bergan et al., 1996[18]). Genistein caused FAK accumulation in the areas of focal cell attachment, and simultaneous complexing between β-1-integrin and FAK was shown to occur without the requirement for FAK activation (Bergan et al., 1996[18]; Liu et al., 2000[18]) (Figure 1(Fig. 1)). Tumor expression of FAK increased, but the levels of activated FAK and the cancer invasion decreased in mice implanted with PC-3-M cells and administered genistein (250 mg/kg of food, 4 weeks) (Lakshman et al., 2008[75]). On the other hand, some results have suggested that genistein application (30 μg/ml, 48 h) decreased the expression of β-1-integrins by 40 % in PC-3 and by 22 % in DU-145 metastatic prostate cancer cells, suppressing the cell adhesion to extracellular matrix elements (Skogseth et al., 2006[120]) (Figure 1(Fig. 1)), which would be the desired effect in target bones, but not at the site of initial dissemination. Such findings call for caution and highlight the importance of correctly timing soy isoflavones application during prostatic cancer disease, so they shouldn't be overlooked in a serious evaluation of their antimetastatic properties.

### Soy isoflavone effects on the ECM-degrading enzymes

Genistein was shown to exert a concentration-dependent inhibitory effect on PSA (fibronectin-degrading proteinase) secretion in androgen-dependent LNCaP metastatic prostate cancer cells. After 5 days of treatment, genistein in a concentration of 100 nM decreased PSA secretion by 25 %; 5 μM of genistein caused a 50 % decrease in the same parameter, while a 90 % reduction of secreted PSA was detected with 50 μM genistein (Davis et al., 2000[34]). In androgen-independent VeCaP metastatic prostate cancer cells, the same duration of genistein treatment induced an inhibitory effect on PSA secretion only at higher (nutritionally irrelevant) concentrations. Namely, the PSA secretion was decreased by 25 % after 10 μM and by 50 % upon administration of 50 μM of genistein, while lower, nutritionally relevant concentrations (0.1-5 μM) were ineffective in this respect (Davis et al., 2000[34]). The basis for the observed decrease in the PSA secretion from these metastatic prostate cancer cell lines represents a genistein-induced multirange inhibition of the PSA gene and protein expression (Davis et al., 2000[34]) (Figure 1(Fig. 1)). Similarly, culturing of LNCaP cells with the presence of soy milk digestion extract (0.79 mg/ml; containing a mixture of genistein, daidzein, glycitein and other isoflavones, whereby ~26 mg/100 g was the concentration of aglycones) significantly reduced the gene expression levels of PSA (Kang et al., 2016[67]). Interestingly, some clinical studies have suggested that prolonged consumption of a soy isoflavone mixture (450 mg genistein + 300 mg daidzein + other isoflavones, daily for 6 months) may not affect PSA levels in prostate cancer patients (deVere White et al., 2010[36]). The crucial impact of soy isoflavones in preventing the ECM degradation and prostate cancer cells expansion is realized through an unambiguously inhibitory effect on the MMP and uPA proteinases (Figure 1(Fig. 1)). Increased expression of MMP-2 at least partly underlies prostate cancer aggressiveness, while the metastatic potential of PC-3 and LNCaP cells coincides with an increased expression of MMP-9 (which is twofold higher in more invasive PC-3 cells) (Upadhyay et al., 1999[133]; Aalinkeel et al., 2004[1]). Genistein has shown a dose- and time-dependent inhibitory effect on the MMP-2 protein expression levels in both LNCaP and PC-3 cells, being the most effective at 50 μg/ml during 48 h (Kumi-Diaka et al., 2006[73]). In line with this observation, MMP-2 activity and gene expression were decreased after genistein application (50 μM, 24 h) in several normal and malignant prostate cell lines (Huang et al., 2005[56]; Xu et al., 2009[137]). In PC-3 cells, genistein (50 μM, 24-72 h) was shown to downregulate MMP-9 activity, gene and protein expression (Li et al., 2006[79]). Equol, applied at concentrations of 10 μM and 50 μM for 24 h, was found to decrease uPA mRNA expression in prostate cancer DU-145 cells, which possess moderate metastatic potential (Zheng et al., 2012[145]) (Figure 1(Fig. 1)).

### Membrane fluidity and invasiveness of prostate cancer cells after soy isoflavones application

**Figure 2:**
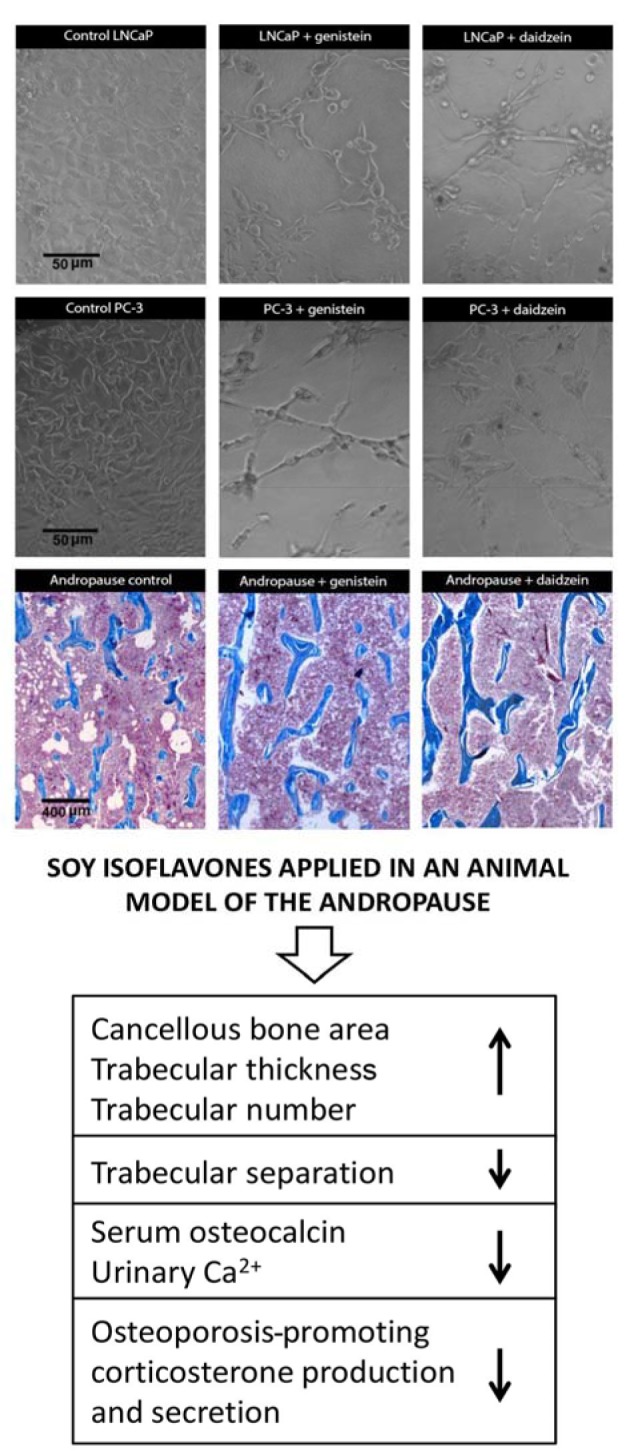
Dynamic phenotype of metastatic prostate cancer cells (LNCaP and PC-3) and the general bone morphofunctional status in an andropausal subject, after treatments with soy isoflavones (Ajdžanović et al., 2009a, b, 2011, 2013, 2014, 2015; Filipović et al., 2010, 2018).

As previously indicated, the increased membrane fluidity of metastatic prostate cancer cells represents one of the intriguing definers of their invasiveness (Ajdžanović et al., 2013[6], 2014[9]). We have demonstrated that short-term exposure to genistein (12.5 μg/ml, 10 min) significantly decreased superficial membrane fluidity in LNCaP and PC-3 cells, which corresponded with the genistein-induced poor invasive trends of these cells in 2.5 D extracellular matrix - Matrigel (Ajdžanović et al., 2013[6], 2014[9]). More precisely, genistein action at the level of the LNCaP cell surface immobilized the membrane androgen receptor containing lipid rafts, downregulated these specific androgen receptors involved in fast signaling from the cell surface and silenced the related downstream pathways (Oh et al., 2010[99]; Ajdžanović et al., 2015[4]) (Figures 1(Fig. 1), 2(Fig. 2)). The effects of genistein on invasive activity and the resulting dynamic phenotype were more prominent in the PC-3 metastatic cell clone, while daidzein, even in higher doses (25 μg/ml), was ineffective when it comes to membrane fluidity and invasiveness of the tested metastatic prostate cancer cells (Ajdžanović et al., 2013[6]; 2014[9]) (Figure 2(Fig. 2)). Considering the fact that the membrane fluidity value of cancer cells is reciprocal to their adhesiveness (Gonda et al., 2010[44]), the effects of genistein observed in this context (adhesiveness promotion) are desirable at the site of initial dissemination, but appear unwanted in distant secondary bone deposits.

### Angiogenesis following prostate cancer growth and soy isoflavones

Along with the soy isoflavone effects that suppress the evolution of metastatic prostate cancer cell malignancy, which is aimed at enabling the cells entry into the bloodstream or lymphatics, their influence on the process of angiogenesis, following the expansion of cancer growth, deserves some attention. Genistein (10-50 μM, 72 h) was shown to significantly inhibit basal and hypoxia-stimulated VEGF gene expression in PC-3 cells (Guo et al., 2007[48]) (Figure 1(Fig. 1)). Subcutaneous inoculation of metastatic prostate cancer LNCaP cells to immuno-deficient mice resulted in reduced cancer mass growth and diminished density of cancer-pervading vessels if a soy phytochemicals-rich diet (containing 341 mg or 1705 mg of isoflavone equivalents/kg) was applied (Zhou et al., 1999[147]). In line with this, a soy isoflavone concentrate (49 % of isoflavones; 200 mg/L, 48 h) reduced the mRNA expression and protein level of the pro-angiogenic cytokine interleukin-8 in PC-3 cells (Handayani et al., 2006[50]) (Figure 1(Fig. 1)).

### Soy isoflavones and the bone aspects of prostate cancer

Metastatic prostate cancer cells adhesion to the vascular endothelium and their extravasation in the areas of future bone lesions may also be obstructed by the use of soy isoflavones. Genistein (10 nM, 24 h) was reported to decrease the gene expression of the endothelial cell adhesion molecule P-selectin (Sandoval et al., 2010[116]), important for the process of association with the prostate cancer cell surface (Figure 1(Fig. 1)). While the character of the inhibitory effects of genistein on the expression of prostate cancer cells-derived, adhesion-locking integrins as well as on the adhesion-defining cancer cell membrane fluidity has already been described above, it should be mentioned here that, in cancer cells, genistein slightly inhibits the mRNA and protein levels of cadherin 11 (Moiseeva et al., 2007[92]), another bone metastasis-promoting marker (Chu et al., 2008[28]). The bone morphofunctional status in prostate cancer patients appears to be important both initially and during treatment, but also independently from the process of metastatic cells invasion (Miñana et al., 2014[91]). Our experimental experience suggests some multidimensional, bone health-related benefits of soy isoflavones application in an animal model of the andropause (orchidectomized, 15-16 months old Wistar male rats). Genistein and daidzein, *s.c.* administrated in a dose of 30 mg/kg b.m., for three weeks, significantly increased the cancellous bone area, trabecular thickness and trabecular number, but decreased the trabecular separation, in the proximal tibial metaphysis of this animal model (Filipović et al., 2010[40], 2018[41]). The molecular mechanisms through which soy isoflavones reinforce the bone microarchitecture in andropausal rats primarily involve ER-dependent pathways (Filipović et al., 2010[40], 2018[41]). In parallel, significant reductions in serum osteocalcin levels and urinary Ca^2+^ concentrations were observed, in comparison with orchidectomized controls (Filipović et al., 2010[40], 2018[41]) (Figure 2(Fig. 2)). Elaboration of the andropausal rat pituitary-adrenocortical axis, under the same conditions, suggested that genistein and daidzein decreased the capacity for production and secretion of corticosterone (Ajdžanović et al., 2009[10][7], 2011[11]) (Figure 2(Fig. 2)), which is important in light of the fact that glucocorticoids are well known to be osteoporosis-promoting factors (Ringe, 1989[109]). Our results confirm numerous other experimental studies reporting beneficial effects of soy isoflavones on the male skeleton in androgen deficiency situations (frequent during prostate cancer therapy; Ishimi et al., 2002[58]; Khalil et al., 2005[69]; Soung et al., 2006[122]), while the related clinical data are still expected.

### Soy isoflavones versus osteolytic prostate cancer metastases

Prostate cancer metastasis-caused osteolytic lesions, which release the space for further malignant growth in the targeted bone, are characterized by significant RANK/ RANKL/OPG signaling (Jin et al., 2011[63]). Genistein (1 g/kg of diet) was found to significantly inhibit the protein expression of RANKL in PC-3-induced tumors in severe combined immunodeficiency (SCID) mice (Li et al., 2006[79]). Genistein and glycitein (10 nM, 14 days) significantly decreased the osteoblast RANKL gene expression *in vitro* (Winzer et al., 2010[135]) (Figure 1(Fig. 1)). Also, genistein (50 μmol/L, 24 h) inhibited the secretion of RANKL protein into the medium in RANKL-transfected PC-3 cells (Li et al., 2006[79]). In line with this, differentiation of RANKL-induced RAW264.7 cells (osteoclast precursor macrophages) to osteoclasts was inhibited by genistein (10 μmol/L) treatment (Li et al., 2006[79]). The complex gene and protein expression as well as the microarray data analysis revealed OPG upregulation in genistein-treated (50 μM, 2-3 days) PC-3 cells (Li et al., 2006[79]). Two times higher levels of OPG mRNA were found in PC-3 bone tumors in SCID mice preventively treated with genistein (1 g/kg of diet, 58 days; Li et al., 2004[78]) (Figure 1(Fig. 1)). In human osteoblastic MG-63 cells, daidzein (0.01, 0.1 and 1 μM, 3 days) increased OPG, but decreased RANKL gene and protein levels, all *via* ER-α and ER-β (Sun et al., 2016[124]). Soy extract (0.001 mg/ml, 6 days) was shown to increase the OPG secretion levels and to decrease those of RANKL in a conditioned medium of MC3T3-E1 osteoblasts (Park et al., 2014[103]). An increased OPG/RANKL ratio was observed in LNCaP and PC-3 cells upon genistein or daidzein (10-50 μM, 72 h) application (Alonso et al., 2009[12]) (Figure 1(Fig. 1)). All these data suggest specific effects of soy isoflavones on concrete constituents of the RANK/RANKL/OPG triad that synergistically strive to deactivate osteoclasts and prevent resorption of the affected bone. A somewhat reserved attitude towards the OPG increase still remains, given its role in the prevention of apoptosis of prostate cancer cells (Holen et al., 2002[54]). In addition, the PTHrP molecule, generally known to elevate the RANKL production in osteoblasts and to decrease the OPG expression and activate osteoclasts in the formation of metastatic lesions (Liao et al., 2008[81]), is susceptible to soy isoflavones actions. Genistein and daidzein (0.01-50 μM, 48 h) were shown to induce the PTHrP gene expression in LNCaP cells, while both isoflavones increased the PTHrP protein expression in these cells even at nM doses during 72 h (Alonso et al., 2009[12]) (Figure 1(Fig. 1)). Actually, these results reflect certain pro-survival effects of soy isoflavones in respect to the metastatic prostate cancer cells (Alonso et al., 2009[12]). Soy isoflavones may directly or indirectly affect TGF-β, the cytokine that also promotes osteolytic metastases (Jin et al., 2011[63]). Gene and protein expression of TGF-β2 in PC-3 cells is reduced upon genistein (50 μM, 6 h, 36 h and 72 h) application (Li and Sarkar, 2002[80]). Isoflavones downregulate the levels of PSA and uPA, TGF-β-activating molecules, in metastatic prostate cancer cells (Davis et al., 2000[34]; Josson et al., 2010[66]; Zheng et al., 2012[145]), which most likely excludes TGF-β from the pool of factors that actively mediate osteolytic metastases when isoflavones are applied.

### Soy isoflavones versus osteoblastic prostate cancer metastases

Metastasis-related osteoblastic lesions *i.e.* foci of irregular bone formation, imply osteoblast proliferation, stimulated by Wnt signaling, through β-catenin-induced gene expression (Behrens et al., 1996[16]). In PC-3 cells, genistein downregulated Wnt-4 protein expression, while the soy isolate mildly decreased β-catenin protein levels (Liss et al., 2010[83]) (Figure 1(Fig. 1)). Genistein-mediated inhibition of IGF-1 (usually upregulated in prostate cancer bone metastasis) also silences the β-catenin pathway (Rubin et al., 2004[112]; Mahmoud et al., 2014[86]). Finally, considering the property of E-cadherin to bind to β-catenin and immobilize it, the increased expression of E-cadherin upon genistein application (15 μM/L, 24 h or 48 h) to prostate cancer cells may suggest some indirect benefits in impeding metastasis (Zhang et al., 2008[144]; Mahmoud et al., 2014[86]).

### Soy isoflavones against the pre-metastatic niche formation: a new horizon?

Until now, the potential of soy isoflavones to influence pre-metastatic niche formation has not been evaluated directly. However, the capacity of these naturally occurring molecules to influence the establishment of pre-metastatic fields in bones, prior to cancer cell arrival, can be discussed in light of the numerous findings confirming their modulatory effect on the mediators involved in this important phase of the cancer dissemination route. One of the factors presumably important in the pre-metastatic ECM bone rearrangement is Hypoxia Inducible Factor 1α (HIF-1α) (Semenza, 2016[117]). Generally, this molecule is the main regulator of the expression of genes critical to cell survival under hypoxic conditions. One of the proteins regulated by HIF-1α is LOX enzyme, a key factor in matrix remodeling in pre-metastatic fields (Joo et al., 2014[64]). Singh-Gupta et al. (2009[119]) found that pretreatment of prostate cancer cells with soy isoflavones downregulated the Stc/STAT3/ HIF-1α pathway and prevented the translocation of HIF-1α into the nucleus. This finding was important from the perspective of soy isoflavones usage in the sensitization of prostate cancer cells to radiotherapy. Yet, the discovery of pre-metastatic fields and the role of HIF-1α at this important stage of the metastatic process have given another important context to the ability of genistein/ daidzein to inhibit HIF-1α nuclear action. Namely, it is known that hypoxic conditions are typical for the bone microenvironment, and together with the hypoxia related high LOX activity, this is one of the main characteristics of the bone tissue matrix (Kingsley et al., 2007[72]). A global quantitative analysis has confirmed that the dominant molecule of the hypoxic cancer secretome, LOX, induces pre-metastatic bone lesions and its appearance is remarkably connected with bone-tropism and relapses (Cox et al., 2015[29]). In view of these circumstances, soy isoflavones may be of use in the prevention of bone metastases, starting from the earliest phase of this process. Also, the soy isoflavone-induced inhibition of RANKL protein secretion into the medium in RANKL-transfected PC-3 cells (Li et al., 2006[79]) and inhibition of RANKL protein expression in a PC-3 xenograft model (Li et al., 2006[79]) have been discussed above. Furthermore, there are numerous sources highlighting the potential of soy isoflavones to directly or indirectly affect the expression and function of TGF-β and, consequently, the pathways regulated by this molecule (Davis et al., 2000[34]; Li and Sarkar, 2002[80]; Josson et al., 2010[66]; Zheng et al., 2012[145]). While RANK/RANKL and TGF-β are on the list of mediators crucial to the establishment of pre-metastatic niches, it is important to note that soy isoflavones can be effective in this (possibly) critical stage for prostate cancer dissemination.

## Conclusions and Evidence-Based Perspectives

**Figure 3:**
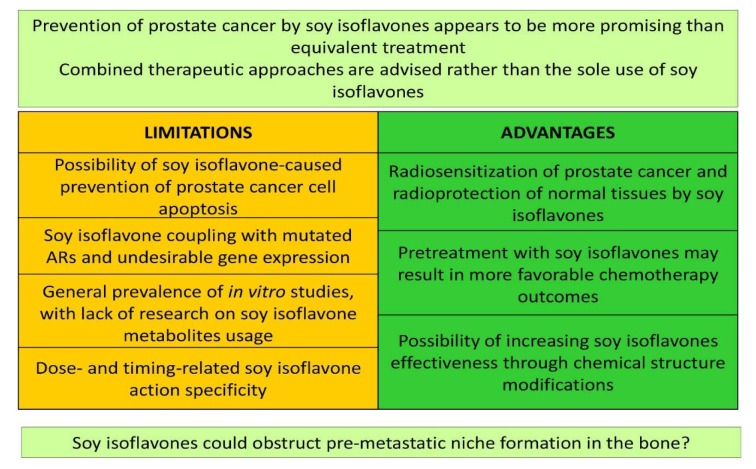
Evaluation of soy isoflavones significance for prostate cancer therapy. ARs - androgen receptors

The impression is that the phrase 'better safe than sorry' is more than adequate in the context of prostate cancer and soy isoflavones, *i.e.*, the prevention of concrete, multivariable malignant disease by using soy isoflavones appears to be more promising than the treatment, especially when the cancer is in a metastatic phase. On the other hand, the applied soy isoflavones have demonstrated beneficial effects at different stages of metastatic prostate cancer progression, including its culmination in the bones. The experience so far indicates that the therapeutic potential of plant-derived compounds is generally exhausted after they have been formulated as dietary supplements/nutraceuticals, and they certainly haven't been positioned as first-line therapeutics for metastatic cancer. Despite certain limitations regarding soy isoflavone actions in prostate cancer cells (prevention of their apoptosis, coupling with mutated ARs, general prevalence of *in vitro* studies that should be extended with the use of isoflavone metabolites) as well as the dose- and timing-related specificity of isoflavones action, the solid evidence of these compounds-induced metastatic sequence disruption (possibly including the aspect of pre-metastatic niche formation) presented herein suggest it might be useful to re-evaluate their therapeutic ranking (Figure 3(Fig. 3)). Thus, soy isoflavones could participate more widely in the combined therapeutic approaches, following the already demonstrated radiosensitization of prostate cancer and radioprotection of normal tissues and organs in the field of radiation, all achieved with the use of soy isoflavones (Raffoul et al., 2007[107]; Ahmad et al., 2010[3]; Hillman, 2019[52]), or given the observed, more effective docetaxel-induced apoptosis of prostate cancer cells pretreated with genistein (Li et al., 2005[77]) (Figure 3(Fig. 3)). However, studies reporting a lack of combination effects of soy isoflavones and taxane chemotherapy on castration-resistant prostate cancer should also be considered (Eskra et al., 2019[39]). The possibility of increasing isoflavone lipophilicity through complexation with transient metal cations and derived inputs on the cell signaling machinery during modified compounds application (Tarahovsky et al., 2014[128]; Ajdžanović et al., 2015[4]) additionally open the gate for therapy fine tuning (Figure 3(Fig. 3)).

## Acknowledgements

This work was supported by the Ministry of Science, Education and Technological Development of the Republic of Serbia, Grant number 173009. Part of the Figure 2(Fig. 2) was adopted from our previous publication and reprinted by permission of the Licensor-publisher Springer (Ajdžanović et al., The Journal of Membrane Biology 246: 307-314, 2013[6]). An appropriate citation is provided in the Figure 2(Fig. 2) legend while the full reference is available in the section “References”. The authors, Vladimir Ajdžanović, Jasmina Živanović and Verica Milošević, participate in the COST Action FA 1403 POSITIVe (Interindividual variation in response to consumption of plant food bioactives and determinants involved), supported by the COST (European Cooperation in Science and Technology). We are grateful to Mrs. Maja Vojvodić, an English language professional, for her help in proofreading the manuscript.

## Conflict of interest

The authors declare that they have no conflict of interest.
